# Is Esophageal Temperature Better to Estimate Brain Temperature during Target Temperature Management in a Porcine Model of Cardiopulmonary Resuscitation?

**DOI:** 10.1155/2017/1279307

**Published:** 2017-12-20

**Authors:** Heng Li, Zhengfei Yang, Yuanshan Liu, Zhixin Wu, Weibiao Pan, Shaohong Li, Qin Ling, Wanchun Tang

**Affiliations:** ^1^Cardiovascular Department, Tung Wah Affiliated Hospital, Sun Yat-sen University, Dongguan, China; ^2^Emergency Department, Sun Yat-sen Memorial Hospital, Sun Yat-sen University, Guangzhou, China; ^3^Emergency Department, Foshan Hospital of Traditional Chinese Medicine, Foshan, China; ^4^Emergency Department, Tung Wah Affiliated Hospital, Sun Yat-sen University, Dongguan, China; ^5^Weil Institute of Emergency and Critical Care Medicine, School of Medicine, Virginia Commonwealth University, Richmond, VA, USA

## Abstract

Brain temperature monitoring is important in target temperature management for comatose survivors after cardiac arrest. Since acquisition of brain temperature is invasive and unrealistic in scene of resuscitation, we tried to sought out surrogate sites of temperature measurements that can precisely reflect cerebral temperature. Therefore, we designed this controlled, randomized animal study to investigate whether esophageal temperature can better predict brain temperature in two different hypothermia protocols. The results indicated that esophageal temperature had a stronger correlation with brain temperature in the early phase of hypothermia in both whole and regional body cooling protocols. It means that esophageal temperature was considered as priority method for early monitoring once hypothermia is initiated. This clinical significance of this study is as follows. Since resuscitated patients have unstable hemodynamics, collecting temperature data from esophagus probe is cost-efficient and easier than the catheter in central vein. Moreover, it can prevent the risk of iatrogenic infection comparing with deep vein catheterization, especially in survivors with transient immunoexpressing in hypothermia protocol.

## 1. Introduction

Mild therapeutic hypothermia (MTH) was regarded as a cornerstone therapy for comatose survivors after the return of spontaneous circulation (ROSC) in instance of cardiac arrest. To maximize the degree of neuroprotection, current guidelines recommended that MTH should be activated with a target temperature of 32–36°C for at least 24 hours [1].

The traditional cooling methods to reduce brain tissue temperature include directly lowering the body temperature and selective head hypothermia [2, 3]. Ideally, target temperature management (TTM) can be guided by brain temperature to maximize neuroprotection, but it is not always feasible. In fact, the pulmonary artery, esophageal, bladder, and rectal temperature can be used to guide the cooling process [4]. Therefore, it is important to control these surrogates of the core temperature during TTM.

However, the “target temperature” management based on core body temperature may be not appropriate for the “target organs,” especially the brain [5]. Improper temperature control might either weaken the neuroprotective effect or interfere with physiological functions. Due to different characteristics of systemic hypothermia and regional cooling, the relationship between brain temperature and surrogates of core temperature, such as pulmonary artery, esophageal, and rectal temperature remains unclear. To achieve precision temperature management in clinical, we designed a randomized, controlled animal experiment to explore the relationship between core temperature and brain temperature under two different hypothermia protocols.

## 2. Materials and Methods

### 2.1. Study Design

This study was approved by the Institutional Animal Care and Use Committee of Sun Yat-sen University. All animals received humane care in compliance with the Guidance Suggestions for the Care and Use of Laboratory Animals, formulated by the Ministry of Science and Technology of the People's Republic of China.

### 2.2. Animal Preparation

Twenty-four domestic pigs weighing from 34 to 36 kg were selected for this study. All pigs fasted overnight except for access to water before the experiment. Anesthesia was initiated by intramuscular injection of ketamine (20 mg/kg), followed by ear vein injection of sodium pentobarbital (30 mg/kg). If necessary, additional doses of sodium pentobarbital (8 mg/kg) were injected hourly to maintain anesthesia. Mechanical ventilation (Model VELA, TBird VELA, California, USA), was supplied, with a tidal volume of 10 ml/kg and inspired oxygen of 0.21. End-tidal P_CO2_ (P_ETCO2_) was monitored via an infrared analyzer (BeneView T5, Mindray Inc., Shenzhen, China). Respiratory frequency was adjusted to maintained P_ETCO2_ at 35~40 mmHg.

Animal preparation was performed following the same procedure as described in our previous study [6]. To obtain arterial pressure (AP), a 6 F catheter (Cordis Brite Tip GC, Bridgewater, NJ, USA) was advanced from right femoral artery to the level of descending aorta. To measure right atrium pressure (RAP) and temperature, a 7 F four-chambered Swan-Ganz catheter (774HF75 Swan-Ganz TD Cather, Edwards Lifesciences Corporation, Irvine, CA, USA) was advanced from right femoral vein and floated to the pulmonary artery. The real-time measurement of *T*_p_ was recorded with the cardiac output analyzer (COM-2, Edwards Critical Care Division, Baxter, Healthcare Corp., CA, USA). For the measurement of *T*_b_, a 5 Fr Swan-Ganz catheter (Arrow International, Inc., Reading, PA, USA) was retrogradely advanced into the cranial cavity from internal jugular vein. For the measurement of *T*_e_, a 14 Fr Silicone Foley Catheter with Temperature Sensor of 35 cm in length (Weil Lead, Medical Corporation, Guangzhou, China) was advanced from the incisor teeth into the esophagus. Rectal temperature (*T*_r_) was measured via a thermal sensor in sigmoid colon. Hard gel defibrillation pads (Stat-padz, Zoll Medical Corporation, Chelmsford, MA, USA) were applied anteriorly to lateral placement.

### 2.3. Experimental Procedure

To electrically induce ventricular fibrillation (VF), a 5 Fr pacing catheter (EP Technologies Inc., Mountain View, CA) was advanced from the right external jugular vein into the right ventricle. A 1 mA alternating current was delivered to ventricle endocardium and maintained untreated VF for 10 minutes. Mechanical ventilation would be discontinued after the onset of VF.

Prior to initiating the resuscitation procedure, the pacing catheter was withdrawn to avoid heart injury during chest compression. After 10 mins of untreated VF, mechanical CPR was initiated. The mechanical chest compressor (Weil SCC™, Sun Life Inc., China) was programmed to provide 100 compressions per minute. The compression depth was adjusted to decrease the anterior-posterior diameter of the chest by 25%. Meanwhile, coinciding with the start of precordial compression, animals were mechanically ventilated with a rate of 10 breaths/min. After 2 mins of CPR, the epinephrine at a dose of 20 *μ*g/kg was administrated to the animals. Five minutes after CPR, a single 120-J biphasic shock (M-Series, Zoll medical corporation, Chelmsford, MA, USA) was attempted to terminate VF. When a rhythm with mean aortic pressure of >50 mmHg persisted for an interval of 5 mins or more, it was regarded as ROSC. CPR was immediately resumed for 2 mins prior to the second round of shock if no ROSC was observed. If there was no evidence of circulation after 10 mins, resuscitation maneuvers would be terminated. ROSC animals were randomized into three groups: selective head cooling group (SHC), intravascular cooling group (IVC), and control groups (Con). In SHC group, nasopharyngeal cooling with the RhinoChill Device (BeneChill, San Diego, CA) was used. The tube was put into the nasal cavity and located into the selective head. The coolant (perfluorodecalin liquid) was pumped into the cavity at the rate of 1 mL/kg/min by compressed oxygen at 1 L/kg/min. The transnasal cooling was initiated when CPR started and maintained for 60 mins, following by intravascular cooling. In IVC group, cooling with intravascular hypothermia (CoolGard 3000, ALSIUS, corporation, Irvine, CA, USA) was initiated during the same time as ROSC. No cooling procedure was carried out for the control group. All animals were maintained TTM for 12 hours with the rewarming rate of 0.5°C.

### 2.4. Measurements

Animal baseline clinical measurements were obtained, including hemodynamic status, blood analysis, and pulmonary core temperature. The ECG, pressure measurements, and acceleration signals were measured and recorded through a data acquisition system supported by WinDaq hardware/software (DATAQ Instruments Inc., Akron, OH, USA) at a sample rate of 300 Hz. The coronary perfusion pressure (CPP) was digitally computed from the differences in time-coincident diastolic aortic and right atrial pressures. Temperature measurements from esophagus, rectum, brain, and pulmonary artery were recorded every 5 mins during postresuscitation (PR) 30 mins and every 10 min from PR 30 min to 360 min.

Quantitative data were presented as mean ± standard variation. Considering there may be autocorrelation for body temperatures in different time point for an individual, we firstly used *T*_b_ as dependent variable, time, and intercept as random effects to fit empty model to explore whether the random effects are significant or not. Secondly, we used *T*_b_ as dependent variable, *T*_p_/*T*_e_/*T*_r_ as independent variable, group, time as covariates, and also time and intercept as random effects to fit linear mixed models. A linear mixed-effect model was used to explore the relationships between *T*_p_, *T*_e_, *T*_r_, and *T*_b_, using *R* squared to evaluate the fitting effects of different models. All analyses were carried out by using SAS PROC MIXED (SAS Institute Inc. NC). A *p* value of <0.05 was regarded as statistical significance.

## 3. Results

### 3.1. Baseline Physiologies and Primary Outcomes of Resuscitation

There were no significant differences in baseline physiologies and primary outcomes of resuscitation among the three groups (Table 1). All animals were successfully resuscitated.

### 3.2. Esophagus/Pulmonary Artery/Brain Temperature Measurement in Three Periods

**Figure 1:**
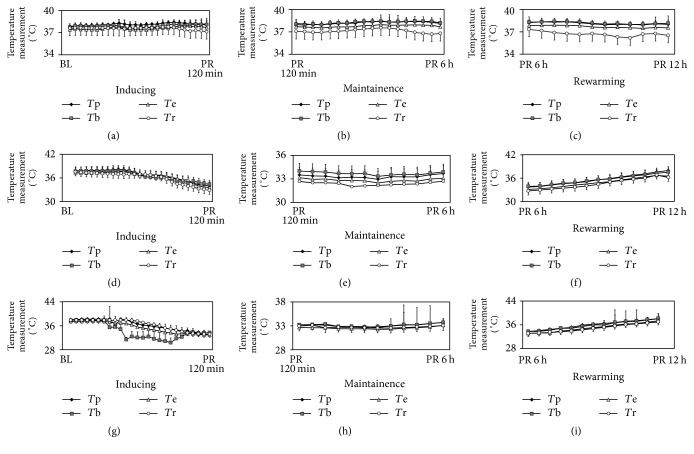
Esophageal (*T*_e_), pulmonary artery (*T*_p_) and brain temperature (*T*_b_) measured in three periods. Normothermia group ((a), (b), and (c)), intravascular cooling group ((d), (e), and (f)), and selective head cooling group ((g), (h), and (i)). Inducing period was defined as time interval from the baseline temperature decrease to 34°C. In our study, time consumed in this period was set to 120 mins. Similarly, maintenance means TTM for 6 hours and rewarming means temperature increased to baseline. While the rewarming rate was controlled at 0.25°C/h, it took another 6 hours to reach the baseline temperature.

*T*_e_ and *T*_b_ slowly decreased in inducing period in IVC group (Figure 1(d)), compared with the abrupt change of *T*_b_ in SHC (Figure 1(g)). It consumed approximately 130 mins for *T*_b_ and 100 mins for *T*_e_ to reach the target temperature of 34°C in both IVC and SHC groups (approximately 0.029°C/min for *T*_b_ and 0.034°C/min for *T*_e_) (Figures 1(e) and 1(h)). In rewarming period, while the rewarming rate was controlled at 0.25°C/h, it took another 6 hours to reach the baseline temperature in both IVC and SHC groups (Figures 1(f) and 1(i)). There was no obvious fluctuation in maintenance period (Figures 1(e) and 1(h)).

### 3.3. Comparisons of Temperature Differences of Rectum/Esophagus/Pulmonary Artery to Brain in Two Hypothermia Groups

**Figure 2:**
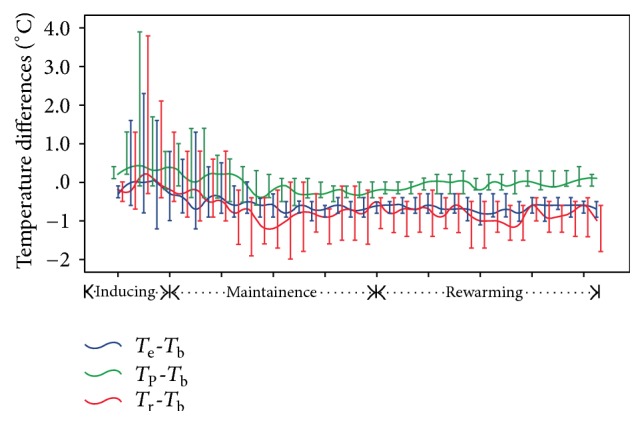
Comparisons of temperature differences of rectum/esophageal/pulmonary artery to brain between hypothermia groups.

In two hypothermia groups (IVG and SHC), the difference among the pulmonary artery, esophageal, and brain temperatures did not exceed 1°C. The largest variability of temperature between groups occurred during the induction period. However, during the maintenance and rewarming period, the difference between the pulmonary artery and brain temperatures varied around zero (Figure 2).

### 3.4. Correlation Analysis between Hypothermia Groups

**Figure 3:**
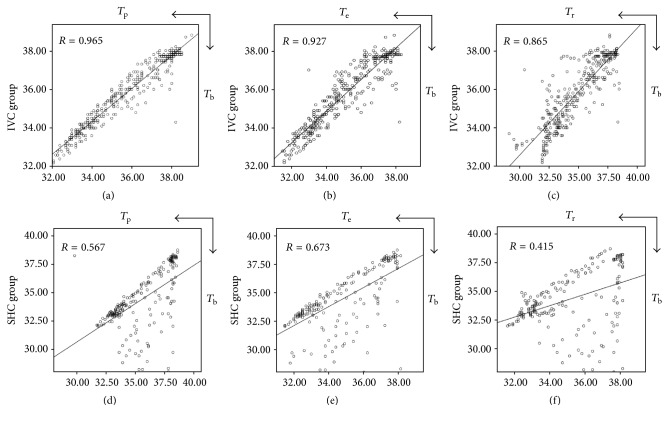
Correlation analysis between hypothermia groups. Correlation of *T*_p_, *T*_e_, and *T*_r_ with *T*_b_ in IVC group (a, b, c). Correlation of *T*_p_, *T*_e_, and *T*_r_ with *T*_b_ in SHC group (d, e, f).

Linear correlation analysis showed that *T*_p_, *T*_e_, and *T*_r_ were strongly correlated with *T*_b_. *T*_p_ had the strongest correlation with *T*_b_ in IVC group. (Figure 3(a), *F* = 4647.56, *p* ≤ 0.001). In SHC group, *T*_e_ and *T*_b_ had the strongest relationship (Figure 3(e), *F* = 163.799, *p* ≤ 0.001), compared with *T*_p_, *T*_r_ that only showed median correlation (Figures 3(d) and 3(f)).

### 3.5. Fit Effects of *T*_r_/*T*_e_/*T*_p_ to *T*_b_ during Hypothermia Groups

With the exclusion of hypothermia periods, esophageal temperature had the highest degree of fitting with brain temperature. More importantly, esophagus temperature also had better indication for brain temperature in the cooling period of induction (Table 2). On the contrary, pulmonary artery temperature could represent brain temperature in following two periods (Table 3).

## 4. Discussion

In this porcine study, we observed that esophageal temperature detected via thermal probe locating at the distal esophagus could better indicate the brain temperature at the time of inducing cooling and maintenance of target mild hypothermia. Regardless of regional or whole-body cooling, esophageal temperature measurement has a great potential as a priority method for TTM.

To maximize preservation of neurological effects, it is now strongly encouraged to induce hypothermia of 32–36°C for comatose victims resuscitated from the shockable arrest [7]. From the physiological point of view, brain temperature should be the priority monitoring site for survivors with TTM. With the fact that the jugular bulb blood is derived from intracerebral vasculature, the jugular vein temperature has been considered as an accurate index of cerebral cortical temperature in various studies [8, 9]. However, both brain cortex and jugular vein temperature are not so easily detected and insufficient data of temperature value may lead to delay initiation and insufficient or excessive TTM.

In these circumstances, appropriated temperature monitoring becomes crucial in the TTM process. The current guidelines recommend the use of core body temperature to guide clinical mild hypothermia [1, 3]. The core temperature measurement is usually collected via standard monitoring sites, such as pulmonary artery, nasopharynx, urinary bladder, tympanic membrane, rectum, and esophagus. Previous studies had evaluated the reliability of temperature measurement in above sites and found that pulmonary artery, urinary bladder, rectal, and esophagus temperature could better reflect the changes of body temperature [10].

Among these different sites of temperature, pulmonary artery temperature is usually regarded as the core body temperature and as a “gold standard” [7]. Thus, when evaluating cooling efficiency, the pulmonary artery temperature has been used as the standard for comparison [11]. However, since induced hypothermia can be initiated immediately after ROSC, it is less realistic to establish vascular catheters when hemodynamic is unstable. As a result, other noninvasive, feasible sites of temperatures are often monitored in place of the pulmonary artery temperature. In clinical practice, temperatures from rectum and axilla were convenient and easy to measured, but their accuracy and stability were still under debate [5].

Esophageal temperature responds rapidly to changes in the temperature of blood perfusing the heart and great vessels and has been recommended as an additional method in TTM [12]. Besides, distal esophageal temperature has been proposed to serve as a reliable index of brain or core blood temperature during hypothermic cardiopulmonary bypass [13].

In our study, we observed that when intravascular cooling was initiated, *T*_p_ had a better correlation with *T*_b_ from the period of maintaining hypothermia to rewarming. Various researchers have supported that the pulmonary artery blood temperature may be the best indicator of brain temperature. Akata et al. found that whenever deep hypothermic cardiopulmonary bypass was started in patients with thoracic aortic aneurysms, the observed degree of accuracy *T*_p_ was superior to other measurements (0.3°C–0.5°C) and its precision superior to other measurements (standard deviation of the difference from *T*_b_ = 1.5°C–1.8°C; correlation coefficient = 0.94–0.95) [14]. Such results can be explained by venous blood of the bronchial circulation returning to the vein and subsequently flowing into the pulmonary artery in a retrograde manner.

However, in selective head cooling after cardiac arrest or in rapid intravascular cooling, pulmonary artery blood temperature indicating brain temperature is challenging. Stone et al. observed that when profound hypothermia was rapidly induced and reversed, temperature measurements measured at standard monitoring sites might not reflect cerebral temperature in patients that underwent cardiopulmonary bypass and deep hypothermic circulatory arrest. However, differences in measurements of nasopharynx, esophagus, and pulmonary temperature could be controlled within 1°C, which might tend to match brain temperature best [15]. A similar animal study was performed by Eshel and Safar, concluding that esophageal temperature during rapid external warming or cooling should be primarily considered [16].

Our study showed that esophagus temperature had a better relationship with brain temperature when inducing hypothermia and early TTM in either selective head or whole-body cooling. We also noted that once TTM was initiated for over 2 hours, the pulmonary artery temperature would have the best reflection of brain temperature. However, no significant differences were observed in both sites of the two hypothermia groups.

This clinical significance of this study is as follows. Since resuscitating and ROSC patients have unstable hemodynamic, collecting temperature data from esophagus probe is cost-efficient, in the sense that it is easier and more rapid to establish than the catheter in central vein [17]. Moreover, the noninvasive esophagus tube prevents the risk of iatrogenic infection comparing with deep vein catheterization, especially in survivors with transient immunoexpressing in TTM.

There were some limitations in our study. First, pentobarbital used for anesthesia may have an adverse impact on brain and cardiac function [18]. Although there was no difference in the dose of pentobarbital among the 3 groups, hypothermia might reduce the metabolism of pentobarbital and affect cerebral activity. Second, the location of the esophageal temperature probe may affect the recorded value. In this study, we placed the temperature probe at a distance of 35 cm from the incisors as a standard measurement location for the esophagus, but because of the possibility of anatomical differences, it might not be ideal for each experimental animal. Third, in animal experiments, we controlled the experimental sample size after obtaining statistical results; our findings require further clinical validation for practical use.

## 5. Conclusion

*T*_e_ had a better correlation with brain temperature in the early phase of TTM in both whole and regional body cooling protocols. It might be considered as a priority method for monitoring temperature during TTM after successful resuscitation.

## Figures and Tables

**Table 1:** Baseline measurement and primary resuscitation outcome.

	Control	IVC group	SHC group
Body wt. (kg)	35.2 ± 1.7	36.3 ± 2.3	36.0 ± 1.1
MAP (mmHg)	114.11 ± 16.42	110.46 ± 10.31	109.84 ± 16.72
*T*_p_ (°C)	37.85 ± 0.35	37.84 ± 0.18	38.02 ± 0.13
CO (L/min)	4.92 ± 0.94	5.00 ± 1.08	4.64 ± 0.57
pH	7.49 ± 0.02	7.52 ± 0.05	7.50 ± 0.05
PCO_2_ (mmHg)	36.38 ± 3.43	35.86 ± 3.50	34.72 ± 4.32
Lactate (mmol/L)	2.23 ± 2.11	2.91 ± 1.32	2.15 ± 0.65
OI	400.79 ± 83.05	458.50 ± 66.02	439.28 ± 51.64
ROSC	8/8	8/8	8/8
Total shocks	2.17 ± 1.47	1.71 ± 1.11	2.00 ± 1.41
Total epi. dose (mg)	1.50 ± 0.55	1.14 ± 0.38	1.25 ± 0.50

Body wt.: body weight; MAP: mean artery pressure; CO: cardiac output; OI: oxygenation index; ROSC: return of spontaneous circulation; epi.: epinephrine. There were no significant differences between three groups.

**Table 2:** Fit effects with *T*_b_ during hypothermia group.

Effect	*R* ^2^	Estimate	Standard error	DF	*t* value	Pr > |*t*|
*T*_p_	0.885	0.728	0.021	611	33.61	<.0001
*T*_e_	0.889	0.783	0.022	611	34.33	<.0001
*T*_r_	0.841	0.575	0.023	611	24.75	<.0001

**Table 3:** Coefficient of determination *R*^2^ with *T*_b_ between three periods.

	Induction	Maintenance	Rewarming
*T*_p_	0.784	0.993	0.990
*T*_e_	0.801	0.992	0.959
*T*_r_	0.750	0.985	0.983

## References

[1] Callaway C. W., Donnino M. W., Fink E. L. (2015). Part 8: Post-cardiac arrest care: 2015 American Heart Association guidelines update for cardiopulmonary resuscitation and emergency cardiovascular care. *Circulation*.

[2] Bernard S. A., Gray T. W., Buist M. D. (2002). Treatment of comatose survivors of out-of-hospital cardiac arrest with induced hypothermia. *The New England Journal of Medicine*.

[3] Nolan J. P., Soar J., Cariou A. (2015). European Resuscitation Council and European Society of Intensive Care Medicine Guidelines for Post-resuscitation Care 2015. Section 5 of the European Resuscitation Council Guidelines for Resuscitation 2015.. *Resuscitation*.

[4] Group H. A. C. A. S. (2002). Mild therapeutic hypothermia to improve the neurologic outcome after cardiac arrest. *New England Journal of Medicine*.

[5] Markota A., Palfy M., Stožer A., Sinkovič A. (2015). Difference between bladder and esophageal temperatures in mild induced hypothermia. *The Journal of Emergency Medicine*.

[6] Peberdy M. A., Callaway C. W., Neumar R. W. (2010). Part 9: post-cardiac arrest care: 2010 American Heart Association Guidelines for Cardiopulmonary Resuscitation and Emergency Cardiovascular Care. *Circulation*.

[7] Robinson J., Charlton J., Seal R., Spady D., Joffres M. R. (1998). Oesophageal, rectal, axillary, tympanic and pulmonary artery temperatures during cardiac surgery. *Canadian Journal of Anesthesia*.

[8] Coppler P. J., Marill K. A., Okonkwo D. O. (2016). Concordance of brain and core temperature in comatose patients after cardiac arrest. *Therapeutic Hypothermia and Temperature Management*.

[9] Yu T., Yang Z., Li H., Ding Y., Huang Z., Li Y. (2015). Short duration combined mild hypothermia improves resuscitation outcomes in a porcine model of prolonged cardiac arrest. *BioMed Research International*.

[10] Kaukuntla H., Harrington D., Bilkoo I., Clutton-Brock T., Jones T., Bonser R. S. (2004). Temperature monitoring during cardiopulmonary bypass - Do we undercool or overheat the brain?. *European Journal of Cardio-Thoracic Surgery*.

[11] Nussmeier N. A. (2005). Management of temperature during and after cardiac surgery. *Texas Heart Institute Journal*.

[12] Poli S., Purrucker J., Priglinger M. (2014). Rapid induction of cooling in stroke patients (iCOOL1): A randomised pilot study comparing cold infusions with nasopharyngeal cooling. *Critical Care*.

[13] Zeiner A., Klewer J., Sterz F. (2010). Non-invasive continuous cerebral temperature monitoring in patients treated with mild therapeutic hypothermia: An observational pilot study. *Resuscitation*.

[14] Akata T., Setoguchi H., Shirozu K., Yoshino J. (2007). Reliability of temperatures measured at standard monitoring sites as an index of brain temperature during deep hypothermic cardiopulmonary bypass conducted for thoracic aortic reconstruction. *The Journal of Thoracic and Cardiovascular Surgery*.

[15] Stone J. G., Young W. L., Smith C. R. (1995). Do standard monitoring sites reflect true brain temperature when profound hypothermia is rapidly induced and reversed?. *Anesthesiology*.

[16] Eshel G. M., Safar P. (1999). Do standard monitoring sites affect true brain temperature when hyperthermia is rapidly induced and reversed. *Aviation, Space, and Environmental Medicine*.

[17] Paik U., Lee T. R., Kang M. (2012). Success rates and procedure times of oesophageal temperature probe insertion for therapeutic hypothermia treatment of cardiac arrest according to insertion methods. *Resuscitation*.

[18] Yang X.-P., Liu Y.-H., Rhaleb N.-E., Kurihara N., Kim H. E., Carretero O. A. (1999). Echocardiographic assessment of cardiac function in conscious and anesthetized mice. *American Journal of Physiology-Heart and Circulatory Physiology*.

